# Insight Into the Laboratory Diagnosis of Periimplantitis Using Reactive Oxygen Metabolite Levels - A Biochemical Study

**DOI:** 10.7759/cureus.41324

**Published:** 2023-07-03

**Authors:** Navina Ravindran, Uma Sudhakar, Nimisha Mithradas, Snophia Suresh, Sherine L Asirvatham, Steffy J, Jhansi L Kotaru, Bakkiya A, Sundaran K. R, Bhavishya B

**Affiliations:** 1 Periodontics, Thai Moogambigai Dental College and Hospital, Chennai, IND

**Keywords:** reactive oxygen species, reactive oxygen metabolite, porphyromonas gingivalis, peri-implantitis, oxidative stress, dental implants

## Abstract

Aim

The study aims to substantiate the quantitative role of the predominant periodontopathogen (*Porphyromonas gingivalis*) associated with peri-implantitis and evaluate the reactive oxygen metabolite levels in peri-implantitis patients.

Methodology

A total of 40 participants were taken from the department of periodontology, Thai Moogambigai Dental College and Hospital, Chennai, and divided into groups I (control) and II (test). Group I included 20 participants with healthy peri-implant tissue, and group II included 20 participants with infected peri-implant tissues. The predominant periodontopathogen was detected by using a quantitative real-time polymerase chain reaction. Samples (gingival crevicular fluid (GCF), saliva, and plasma) were collected, and a biochemical assay was conducted for reactive oxygen metabolites (ROM) analysis in healthy implants (control group) and peri-implantitis conditions (test group). ROM levels of the patients were statistically analyzed.

Results

The qualitative and quantitative profiles of *Porphyromonas gingivalis* (*P. gingivalis)* associated with Peri-implantitis were analyzed, and the levels of ROM in periimplantitis patients were assessed. The study results substantiate the quantitative picture of *Porphyromonas gingivalis* in the detection of periimplantitis. The saliva and GCF samples showed significant differences in ROM levels between the test and control groups.

Conclusion

This is one of the few studies to detect the predominant bacterial pathogen associated with peri-implantitis and assess the ROM levels in periimplantitis patients. The study gives a correlation between the periopathogens and ROM levels, thereby facilitating the attainment of the best possible treatment options.

## Introduction

Dental implants have gained popularity in recent years as they provide a reliable, cost-effective, and dependable solution for the replacement of missing teeth. The increasing demand for dental implants over traditional methods of tooth replacement has seen a rise in post-treatment complications. Failure of dental implant integration can occur either after immediate implant placement, during ridge healing, or long after initial placement. Several factors contribute to the failure of dental implants, such as inadequate bone volume or quality, occlusal overload, micromovement during healing, foreign body type immunologic rejection, and bacterial colonization [1]. These major complications associated with post-implant surgery are due to the inflammation caused by bacterial invasion [2-4]. Consequently, treating peri-implant infection and related problems is becoming a significant factor in the long-term prognosis of dental implants.

Peri-implantitis (PI) is one of the most common complications seen after dental implant placement. Its pathogenesis involves the invasion of pathogens in the peri-implant tissue leading to inflammation and loss of supporting structures. Currently, two clinical conditions are seen; (i) peri‐implantitis and (ii) peri‐implant mucositis. The clinical and histopathological changes associated with the alteration of peri‐implant mucositis to peri‐implantitis are not clearly appreciated. Although the presence of inflammation is a key feature in both conditions, only peri-implantitis is associated with the loss of supporting bone structure [5]. The peri‐implantitis disease progression occurs in a non‐linear pattern, with the onset occurring at the early stages of follow‐up.

Implant surface exposure to the oral cavity leads to the formation of biofilm. The biofilm acts as an interphase between the implant surface and the initial colonizer, such as *Streptococcus mitis*, *Streptococcus sanguis,* and *Streptococcus oralis*. The initial colonizers create an environment for the adhesion of periodontopathogens, which induces peri-implantitis [6]. In a few cases of peri-implantitis, periods of rapid destruction are noted along with changes in host response which requires timely management [7]. Peri-implantitis, which is a common complication of dental implant procedures, occurs frequently; therefore, insight into the diagnosis of the microorganisms that influence the peri-implant tissue is needed for carrying out various treatment options.

An uncontrolled chronic inflammatory response caused by polymorphonuclear neutrophils (PMNs) can be seen in the case of periodontitis, and similar conditions can be noted in peri-implantitis conditions. PMNs play a major role in host defense mechanisms and also the wound healing process after tissue injury. The oxidative stress induced by reactive oxygen species (ROS) produced by PMNs can destroy potential pathogens and can also cause host tissue damage. Lack of resolution of inflammation in periodontitis and peri-implantitis condition leads to activation of PMN, tissue damage, excessive scarring, improper wound healing, and finally leading to failure of the treatment procedure [8-10]. 

An increase in reactive oxygen species (ROS) level is one of the key pathological parameters noticed in periodontal lesions. Normal oxygen metabolism produces a natural by-product ROS, which plays an important role in cell-to-cell signaling and tissue homeostasis. Environmental stress leads to an increase in ROS levels and results in serious tissue damage. In the peri-implantitis condition, the oral bacteria induces the host cell neutrophils to release ROS as a host immune response [11]. Therefore, the present study aims to detect the predominant periodontal pathogen-associated with peri-implantitis and to evaluate the reactive oxygen metabolite levels in peri-implantitis patients.

## Materials and methods

The present study is a case-control study performed in the outpatient pool of the department of periodontology, Thai Moogambigai Dental College and Hospital, Chennai. The Institutional Ethical Committee of Dr. MGR Educational and Research Institute approval was obtained (Dr.MGRDU/TMDCH/2017-2018/24012011). Informed consent from the patients was obtained.

A total of 40 participants were taken in this study and divided into group I (control) and group II (test). Group I had 20 participants with healthy peri-implant tissue, and group II had 20 participants with peri-implantitis (PI). In PI cases, those implants with probing depth (PD) ≥5 mm, with bleeding on probing (BOP), and radiographic bone loss involving ≥2 threads were taken for the study. Implant sites with the absence of BOP, PD ≤3 mm, and no radiological bone loss was taken as healthy implant controls (HIs). Previously periodontally treated patients, systemically unhealthy patients, those with intake of antibiotics in the preceding six months, smokers, and pregnant or lactating females were excluded.

Clinical parameters and radiographic examination

**Figure 1:**
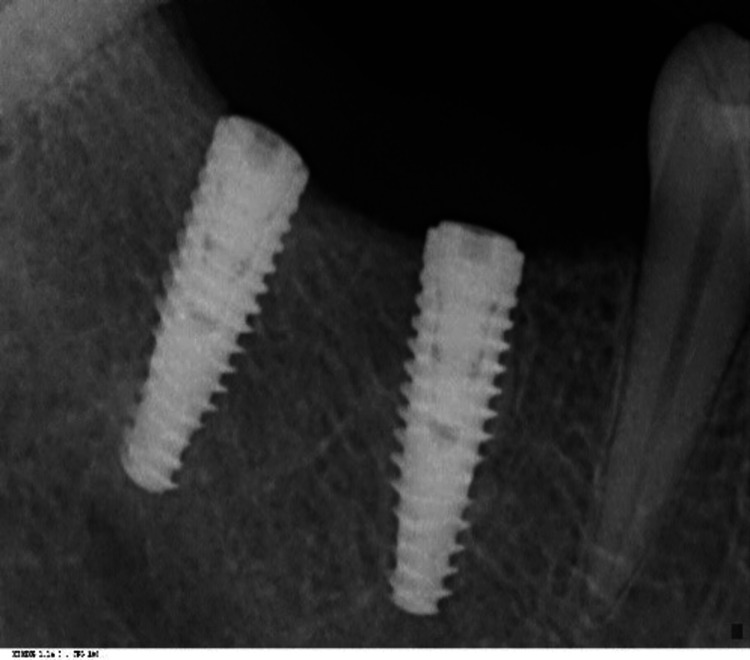
Radiograph of healthy implant site (control group) The radiograph depicts an implant with a normal bone level and an absence of radiolucency around the implant site.

**Figure 2:**
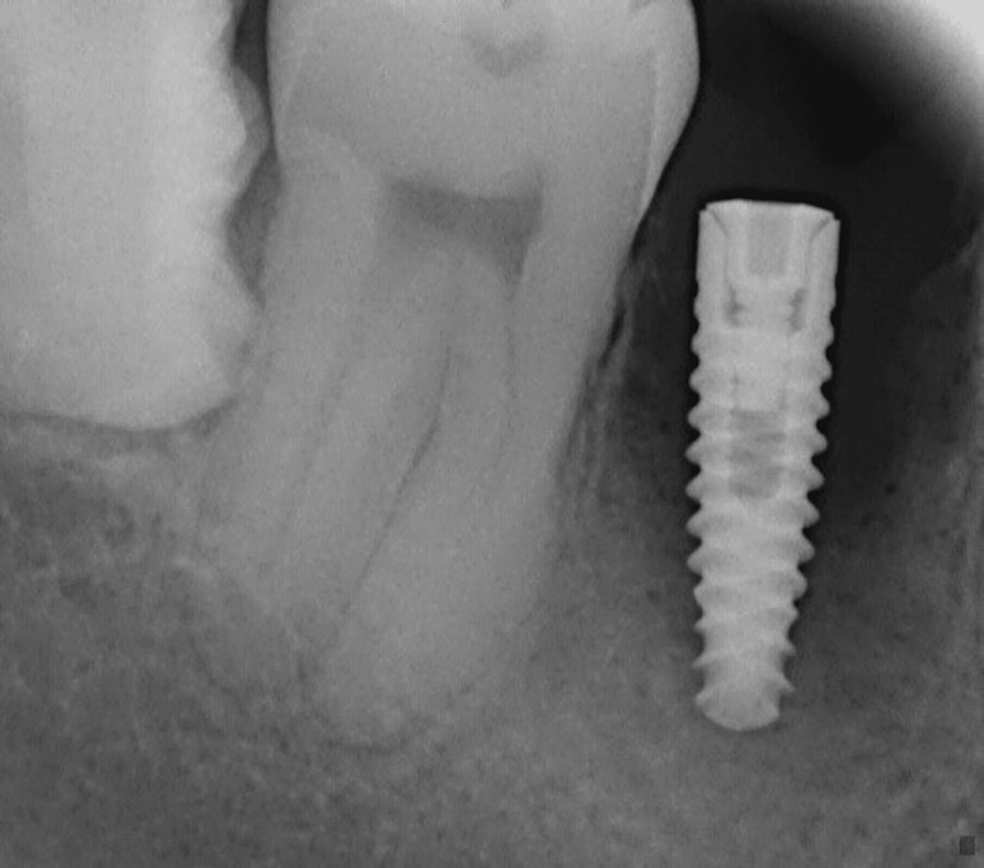
Radiograph of peri-implantitis site (test group) The radiograph depicts an implant with marked bone loss and the presence of radiolucency around the implant site.

The following clinical parameters at six sites per implant using a periodontal probe graded in mm (University of North Carolina 15) were taken: (1) clinical probing depth in mm; (2) dichotomous bleeding index by Ainamo and Bay (15 seconds after probing) [12]; (3) dichotomous plaque index by Dienzer [13]. Radiographs were taken for both the control and test groups (Figures 1, 2)

Microbiological sampling

The subgingival plaque samples were collected, and a quantitative real-time polymerase chain reaction (RT-PCR) was performed. The presence or absence of *Porphyromonas gingivalis* (Pg) was detected and quantified.

Collection of samples

A standardized volume of 1μl gingival crevicular fluid (GCF) was collected from each site with an extra-crevicular approach. The GCF was changed to Eppendorf tubes and stored (-70 C). Blood (3 ml) was centrifuged at 3000 ×g for five minutes. If not assayed immediately, the plasma aliquots were stored at -80ºc. Unstimulated saliva samples were collected by a draining/ spitting method into a graduated container.

Laboratory method for detection of ROM

The laboratory detection of ROM levels was done in the Regenix Super Speciality Laboratory, Chennai. The determination of reactive oxygen metabolite (d-ROM) test is a photometric test that allows assessment of the pro-oxidant status in biological samples by measurement of the concentration of hydroperoxides (ROOH) via the Fenton reaction. ROOH in cells shows the oxidative attack of ROS on various organic substrates.

Statistical analysis

The comparison of bacterial loads between peri-implantitis patients and healthy participants was made using the Mann-Whitney test. A comparison of the mean ROM levels between PI and healthy controls in GCF, plasma, and saliva was made using Anova. The statistical analysis was performed using commercial software SPSS version 20.0 (IMB Inc., Armonk, New York) with the significance level α = 0.05.

## Results

A total sample of 40 participants was divided into group I (control) and group II (test). Group I included 20 participants with healthy peri-implant tissue, and group II included 20 participants with peri-implantitis.

Table 1 reports differences in bacterial loads of *Porphyromonas gingivalis* (*P. gingivalis*) between healthy peri-implant tissue (control group) and peri-implantitis patients (test group).

**Table 1:** Comparison of bacterial loads of Porphyromonous gingivalis between group I (healthy) and group II (peri-implantitis)

Porphyromonous gingivalis level (μm)	Mean	Standard deviation	P-value
Group I (Healthy)	13.24	0.126	1.000
Group II (Peri-implantitis)	13.25	0.093	

The levels of *P gingivalis* were significantly increased in peri-implantitis when compared with healthy patients. Table 2 reports the comparison of reactive oxygen metabolite (ROM) levels in peri-implantitis and healthy participants in saliva, gingival crevicular fluid, and plasma.

**Table 2:** Comparison of mean reactive oxygen metabolite (ROM) levels between group I and group II in gingival crevicular fluid (GCF), plasma, and saliva samples

Reactive oxygen metabolite (ROM) levels - Carratelli units (CARR U)		Mean	Standard deviation	P-Value
Gingival crevicular fluid (GCF)	Group I	274.35	51.84	<0.001
	Group II	532.53	65.94	
Plasma	Group I	287.04	48.14	0.13
	Group II	577.53	56.88	
Saliva	Group I	299.17	47.35	<0.001
	Group II	592.07	54.44	

The study results show a statistically significant difference in the ROM levels in PI and healthy implants in GCF and saliva samples, whereas no statistically significant difference was observed in plasma samples. 

## Discussion

The results of the present study show that the qualitative analysis of *Porphyromonas gingivalis* between group I and II was not different. However, the quantitative analysis of perio-pathogen showed higher levels in PI. This was in accordance keystone pathogen hypothesis of periodontal disease, which emphasizes that the presence of perio-pathogens between disease and health is similar, but their quantitative analysis indicates the disease state [14].

Research has been done earlier, focusing on changes in the microbial picture of stable and failing implants. Studies have proved that the microbiota around healthy implants is indistinguishable from those around healthy teeth. Similarly, failing implants have those similar to the microbiota around periodontitis. However, the microbiota around failing implants is based on the type of implant failure. The present study substantiates that the quantitative profile of the microorganism plays a major role in the disease progression.

The current study is one of the few studies that evaluate the level of reactive oxygen metabolites (ROM) in peri-implantitis patients. The imbalance between reactive oxygen species (ROS) and antioxidants lead to inflammatory changes in the periodontium [15]. In the current study, the plasma ROM levels in peri-implantitis and healthy patients did not differ significantly. Whereas the ROM levels were significantly increased in saliva and GCF samples, with the highest value seen in GCF samples similar to studies done by Sobanice et al. [16], Sheikhi et al. [17], Tuter et al. [18]. The rise in ROM levels in saliva can be noted either due to leakage of ROS from plasma to saliva or due to attack of ROS in saliva against the invading pathogens [19].

The higher level of ROM in GCF compared to saliva and plasma can be due to a site-specific increase, which was notably more compared to a systemic increase in the pathogenesis of periodontal disease [20]. Few of the GCF elements and metabolites of tissue degradation were seen in the unstimulated saliva aiding in the determination of the tissue degradation process [21].

The present study was done with a smaller sample size which could be a limitation. Further, longitudinal studies with larger sample sizes could be helpful in determining the role of reactive oxygen metabolites in peri-implant health and disease states. Also, studies need to be done to get an overall bacterial picture for easy early diagnoses and to carry out effective treatment modalities.

## Conclusions

The results of the current study give an insight into the bacterial load present at the peri-implantitis site. Further, this study suggests that significant oxidative stress may occur in peri-implantitis, which can be contributing factor for increased peri-implant tissue pathogenesis and tissue degradation. 
